# Visual Pretraining via Contrastive Predictive Model for Pixel-Based Reinforcement Learning

**DOI:** 10.3390/s22176504

**Published:** 2022-08-29

**Authors:** Tung M. Luu, Thang Vu, Thanh Nguyen, Chang D. Yoo

**Affiliations:** School of Electrical Engineering, Korea Advanced Institute of Science and Technology, Daejeon 34141, Korea

**Keywords:** representation learning, vision-based deep reinforcement learning, deep reinforcement learning, sample efficiency

## Abstract

In an attempt to overcome the limitations of reward-driven representation learning in vision-based reinforcement learning (RL), an unsupervised learning framework referred to as the visual pretraining via contrastive predictive model (VPCPM) is proposed to learn the representations detached from the policy learning. Our method enables the convolutional encoder to perceive the underlying dynamics through a pair of forward and inverse models under the supervision of the contrastive loss, thus resulting in better representations. In experiments with a diverse set of vision control tasks, by initializing the encoders with VPCPM, the performance of state-of-the-art vision-based RL algorithms is significantly boosted, with 44% and 10% improvement for RAD and DrQ at 100 steps, respectively. In comparison to the prior unsupervised methods, the performance of VPCPM matches or outperforms all the baselines. We further demonstrate that the learned representations successfully generalize to the new tasks that share a similar observation and action space.

## 1. Introduction

Recent advances in deep reinforcement learning (RL) have allowed agents to perform complex control tasks directly from raw sensory observations. Remarkable successes have been achieved, ranging from learning to play video games from raw pixels [1], solving complex tasks from first-person-view observations [2,3], to autonomously performing robotic tasks [4,5,6,7].

As a standard practice, deep RL methods jointly learn a visual encoder and a policy in an end-to-end manner. In this paradigm, the visual representations are learned under the strong supervision of task-specific rewards. While the simplicity of end-to-end methods is appealing, learning the representations relying on the rewards can have several limits. First, the representations are acquired harder under sparse rewards, thus requiring more data for convergence. Additionally, in practice, the reward function is commonly designed and retested until a suitable function is selected, resulting in the representation learning process being repeated, thus it is inefficient. Furthermore, whenever the agent encounters the new tasks, the representation learning is performed again even if the environment appearance is identical across tasks.

**Figure 1:**
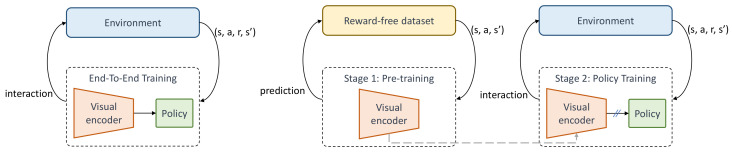
The end-to-end training paradigm (**left**) and our two-stage training (**right**). In the end-to-end training, the visual encoder is jointly trained with the policy under the supervision of rewards from the environment. In contrast, our method detaches the representation learning from the policy learning. In the first stage, the visual encoder is trained with the proposed contrastive predictive model without rewards and is frozen. Then, in the second stage, given some tasks, the policy is trained by reusing the frozen encoder.

In this work, we pursue an alternative paradigm as depicted in Figure 1, wherein the visual encoder is first pretrained without any reward supervision, detached from the policy learning, and then the learned task-agnostic representations are transferred for learning policy on a specific task, under the reward supervision. This two-stage training enables the representations learned in an environment to be reused for other tasks that use the same environment, such as the cheetah environment, containing moving forward and backward tasks. Furthermore, the policy learning stage also requires fewer samples for training, and is thus more sample efficient.

In the first stage, a natural choice is pretraining the visual encoder on a large dataset such as ImageNet. However, previous works show that naively pretraining in such a dataset does not lead to a significant impact [8,9,10]. This ineffectiveness might stem from the domain discrepancy, and more importantly, the pretraining data is lacking in reflecting natural dynamics relation, i.e., the Markov decision process (MDP) property, which is inherent in RL tasks. As we show later in our experiments, pretraining the visual encoder with even in-domain data without considering dynamics is still underperformed. Early works [11,12,13] commonly learn the compact representation by reconstructing the pixels in the current or subsequent frame, which is very challenging with high-dimensional observations. Recently, ref. [14] introduced the contrastive prediction to bypass the use of reconstruction-based prediction. However, this method is lacking in capturing the environment dynamics, thus resulting in low performance in complex tasks such as Cheetah or Walker. To mitigate this shortcoming, ref. [15] introduced augmented temporal contrast (ATC), which additionally trains a marginal forward dynamic model as an auxiliary task for learning representation. Despite effectiveness, the proposed marginal forward model does not condition actions while learning, thus making ATC focus on temporal coherence between observations rather than modeling environment dynamics. To address these issues, this paper introduces a method referred to as the visual pretraining via contrastive predictive model (VPCPM). Specifically, VPCPM utilizes the reward-free data from the environment to learn the visual encoder by jointly optimizing the forward and inverse dynamic models through the contrastive objective. The visual encoder firstly maps the observation into the latent state. Given a latent state and a latent action, the forward model predicts the next latent state. Meanwhile, the inverse model infers the executed action given the consecutive latent states. Learning the forward and inverse models serves as the constraint to enable the visual encoder to consistently follow the underlying dynamics of the environment.

The proposed method is evaluated on a diverse set of image-based continuous control tasks from DeepMind Control Suite [16]. The experiment results show that VPCPM consistently improves the performance and sample efficiency of the state-of-the-art vision-based RL algorithms. Specifically, at 100k environment step, VPCPM improves the mean returns 44% over reinforcement learning with augmented data (RAD) [17], 10% over data-regularized Q (DrQ) [18]. Compared to prior unsupervised pretraining methods, VPCPM matches or outperforms all the baselines as tested across all environments. Moreover, the investigation of unseen tasks shows that the VPCPM-initialized encoder successfully generalizes to unseen tasks that operate in the same environment.

The rest of the paper is organized as follows: Section 2 discusses related works, while Section 3 describes setup in vision-based RL and the related base algorithm. Section 4 details our proposed method. Section 5 presents extensive experiments of the proposed methods, while the conclusion of our paper is presented in Section 6.

## 2. Related Work

**Vision-based Deep Learning.** Since the breakthrough results of convolutional neural network (CNN) [19] in the 2012 ILSVRC image classification task, the deep learning approaches have dominated in almost every recognition and detection task. Many works have already implemented the vision-based deep learning methods to detect the number and location of occupants [20,21,22], perform instance segmentation from RGB images [23,24,25] or point cloud [26,27,28], and reconstruct 3D scenes [29,30,31]. In robotics control, by leveraging the expressive CNN together with the advanced reinforcement learning methods, there are many works that successfully perform the complex manipulation tasks based on visual perception [6,7,32,33]. Despite these successes, there are still challenges lying ahead in achieving versatile, robust, and generalized representations.

**Representation Learning for RL.** Several methods have been proposed in the literature to learn better representations for vision-based RL. Ref. [2] proposed auxiliary tasks including next-observation and reward-based prediction in improving the performance on DMLab [34]. Representation learning using the reconstruction-based task is also a common method, where a beta variation autoencoder (*β*-VAE) [35] or a deterministic AE is applied for learning a low-dimensional representation, and subsequently, a policy was learned using this representation. Recent work [36] attempted to jointly learn VAE/AE together with RL objective, while [12] proposed to train the two objectives one after the other in an alternating fashion. Ref. [13] also used the VAE for learning the representation in the goal-conditioned setting. These methods only focus on learning the representation of an observation rather than modeling the environment dynamics. Moreover, the use of reconstruction-based tasks makes algorithms hard to scale in high-dimensional environments.

Leveraging the predictive model, which is the combination of the forward and inverse dynamic models, for learning representation is also commonly used. Ref. [37] constructed a joint inverse-forward model to learn representation, in which the forward model is used as a regularizer for training the inverse model features. Ref. [38] used only the inverse model to learn the representations, and then the learned representations were used for the forward model to compute intrinsic reward. Ref. [39] learned the representation by combining the forward and inverse models using cycle consistency losses. In this approach, the use of the forward model makes the representations more Markovian [40,41], i.e., the next latent state only depends on the current latent state and action. In the meanwhile, the inverse model encourages the encoder to capture the controllable features. However, this also makes the representation ignore the uncontrollable features that cannot be captured by the inverse model, which might be useful for solving tasks. Our method proposes the use of contrastive loss while learning the forward model to prevent this degeneracy.

Several methods leverage the recent advances in self-supervised learning for acquiring the representation [42,43,44,45,46] in order to improve the performance of the base RL algorithms. Ref. [42] applied a variant of the noise-contrastive estimation loss on future steps on top of A2C [47] to learn better representation and improve sample efficiency, although the results were limited. In the Atari [48] domain, ref. [49] introduced a new contrastive loss to learn better representation in a fully unsupervised setting, while in [45], the authors adapted the BYOL objective [50] for learning the visual encoder in parallel with the RL objective, which led to state-of-the-art results on the Atari benchmark. In the vision-based continuous control domain, ref. [14] introduced a general framework that is a combination of contrastive loss with image augmentation for learning the representation in a more sample efficient way. Ref. [46] proposed a contrastive version of the conditional entropy bottleneck objective [51] to learn a compressed representation of the predictive information of the environment dynamics. Furthermore, to learn the robustness representation, ref. [52,53] attempted to embed the bisimulation metric [54] into the latent space to encourage the representations invariant to the distractors and generalized to unseen environments. Recently, refs. [17,18] showed that a modest degree of image augmentation can significantly improve sample efficiency for learning directly from visual observation. Most of the existing methods learn the visual encoder by optimizing the unsupervised/self-supervised auxiliary and the RL objective simultaneously, which can be considered as learning from scratch, in an end-to-end manner. By contrast, our framework considers training in two stages, and we further propose a new self-supervised auxiliary objective in the pretraining stage to provide a meaningful representation for directly using or further fine-tuning in the testing environment.

**Unsupervised Pretraining Representation for RL.** Pretraining the representations without supervision using an unsupervised or self-supervised framework is a common practice in other fields, such as natural language processing (NLP) [55,56,57] or computer vision [42,43,44,50,58,59]. These studies consider effective ways to learn the visual encoder from massive unlabeled data and reduce sample complexity when learning a new task. In deep RL, early studies attempted to pretrain the visual encoder by using pixel-reconstruction task [11,12] or object detection task [60]; the pretrained representations are then fine-tuned on a specific task. Some recent studies [8,9,10] showed that naively pretraining on ImageNet is not helpful for performing the downstream RL tasks. It hints that the visual encoder of the RL agent would be pretrained on the data closely related to its environments. Along this line of research, ref. [14] attempted to use their proposed objective, originally designated for online training, to learn the visual encoder detached from policy learning. However, due to the lack of encoding of the environment dynamics, the performance was limited in complex tasks such as Cheetah. The study closest to ours is [15], which also attempted to learn the encoder detached from the RL objective. However, this method implicitly estimates the next observation by marginalizing over actions. In contrast, our method relies on the forward and inverse dynamics models with contrastive loss, where the proposed forward model is conditioned on action such that in the latent space, the representation of states and action consistently follow the underlying Markov decision process of the environment.

In addition to pretraining the visual encoder, there is also another line of research that tries to pretrain the policy with self-supervised intrinsic rewards [38,61,62,63,64,65]. In this setting, the agent is first allowed to freely interact with the environment for a long period without access to extrinsic rewards, then it is exposed to task-specific rewards to learn on downstream tasks. The intrinsic reward is commonly formulated to encourage the RL agent to gain new knowledge about the environment [38,61,62], maximize diversity of collected data [9,66], or learn diverse skills [67,68,69,70]. For the vision-based RL tasks that are applicable in this setting, the visual encoder is concurrently trained with the policy during pretraining. Recently, ref. [9] proposed to use a particle-based estimator [71] to estimate entropy for observations, with its representations learned by using contrastive loss from SimCLR [44]. Alternatively, ref. [66] proposed a self-supervised pretraining scheme that allows detaching the representation learning from exploration (i.e., learning from intrinsic rewards) to enable the generalization of representations for unseen tasks. In this method, the representations are learned by a variant of clustering-based contrastive loss SwAV [72]. These works are promising to acquire the general policy as well as the generalized representations. However, they are still required to freely interact with the environment during pretraining, which is potentially unsafe in the real world. In contrast, our framework allows us to learn the encoder entirely from an offline dataset, thus resulting as safer and enabling the reuse of past data.

## 3. Background

In this section, the framework for vision-based reinforcement learning is presented together with a representative off-policy model-free algorithm, soft actor-critic.

### 3.1. Reinforcement Learning from Images

The problem of solving control task from high-dimensional observations is formulated as a partially observable Markov decision process (POMDP) [73,74], which can be defined as a tuple (O,A,p,r,γ). Here, O is the high-dimensional observation space, A is the action space, the transition dynamics $p=Pr(o_{t+1}|o_{\leq t},a_{t})$ represent the probability distribution over the next observation $o_{t+1}$ given the history of previous observations $o_{\leq t}$ and current action $a_{t}$, the reward function r:O×A→R that maps the current observation and action to a reward $r_{t}=r\left(o_{t},a_{t}\right)$, and γ∈[0,1) is a discount factor. Following common practice [1], the POMDP is reformulated as an MDP [73] by stacking consecutive observations into a state $s_{t}=\left\{o_{t},o_{t-1},o_{t-2},\ldots \right\}$. For simplicity of notation, the transition dynamics and reward function are redefined as $p=Pr(s_{t+1}|s_{t},a_{t})$ and $r_{t}=r\left(s_{t},a_{t}\right)$, respectively. The goal of RL is to find a policy $\pi (a_{t}|s_{t})$ that maximizes the expected return defined as the total of accumulated reward $E_{\pi }\left[\sum_{t=0}^{T}\gamma^{t}r_{t}|a_{t}∼\pi \left(.|s_{t}\right),s_{t+1}∼p\left(.|s_{t},a_{t}\right),s_{0}∼p_{0}\left(.\right)\right]$, where *T* is the length of episode and $p_{0}$ is the probability distribution of initial state.

### 3.2. Soft Actor-Critic

Soft actor-critic (SAC) [75] is an off-policy actor-critic method based on the maximum entropy RL framework [76], which encourages the exploration and robustness to noise by maximizing a weighted objective of the reward and the policy entropy. To update the parameters, SAC performs the soft policy evaluation and improvement steps. The soft policy evaluation step fits a parametric Q-function $Q(s_{t},a_{t})$ using transitions from the replay buffer D by minimizing the soft Bellman residual:

(1) $$J\left(Q\right)=E_{(s_{t},a_{t},r_{t},s_{t+1})∼D}\left[\left(Q\left(s_{t},a_{t}\right)-r_{t}-\gamma \bar{V}{\left(s_{t+1}\right)}^{2}\right)\right].$$

The target value function $\bar{V}$ is approximated via a Monte Carlo estimate of the following expectation:

(2) $$\bar{V}\left(s_{t}\right)=E_{a_{t}∼\pi }\left[\bar{Q}\left(s_{t},a_{t}\right)-\alpha \log\pi \left(a_{t}|s_{t}\right)\right],$$

where $\bar{Q}$ is the target Q-function, with its parameters obtained from an exponentially moving average of the Q-function parameters for stabilizing training. The soft policy improvement step then updates the stochastic policy π by minimizing the following objective:

(3) $$J\left(\pi \right)=E_{s_{t}∼D}\left[E_{a_{t}∼\pi }\left[\alpha \log\pi \left(a_{t}|s_{t}\right)-Q\left(s_{t},a_{t}\right)\right]\right].$$

In this work, the learnable version of temperature *α* is used instead of the pre-fixed value, which is optimized by the following objective:

(4) $$J\left(\alpha \right)=E_{(s_{t},a_{t})∼D}\left[-\alpha \log\pi \left(a_{t}|s_{t}\right)-\alpha \bar{H}\right],$$

where $\bar{H}\in R$ is the target entropy hyperparameter that policy attempts to match, which in practice is usually set equal to -|A|.

SAC is one of the state-of-the-art RL algorithms for continuous control [75]. It is also widely used as a backbone for solving vision-based control tasks [5,14,17,18,36,46]. In this work, we adopt RAD [17] and DrQ [18], which are built on top of SAC, for policy learning.

## 4. Method

In this section, the proposed visual pretraining via contrastive predictive model (VPCPM) is described. The proposed method can be used to pretrain the visual encoder, which is then utilized for policy learning by common model-free vision-based RL algorithms.

### 4.1. Network Architecture

The control policy network takes the input as the state $s_{t}$ and outputs the action $a_{t}$. It consists of the visual encoder $\pi_{e}$ parameterized by ϕ and the policy $\pi_{a}$ parameterized by $\theta_{a}$, as depicted in Figure 1. This design enables the encoder to be trained independently with the policy part, i.e., without requiring the RL objective for training. The goal of the proposed method is to learn useful representations from an amount of given data without rewards, such that $\pi_{a}$ can be efficiently trained on top of that to solve RL tasks.

### 4.2. Visual Pretraining via Contrastive Predictive Model

VPCPM introduces a useful prior for the vision-based RL training procedure by enforcing the representations not only representing the semantic information but also conforming to the basis of dynamics from the environment. During the pretraining stage, for a given environment, it is assumed that there is a pre-collected dataset D consisting of *N* transitions without task-specific rewards: $\left(s_{t}^{\left(i\right)},a_{t}^{\left(i\right)},s_{t+1}^{\left(i\right)}\right)$ with the index i={1,…,N}. The visual encoder is desired to effectively encode the semantics and consistently follow dynamics only from the primitive elements, i.e., observations and actions.

**Figure 2:**
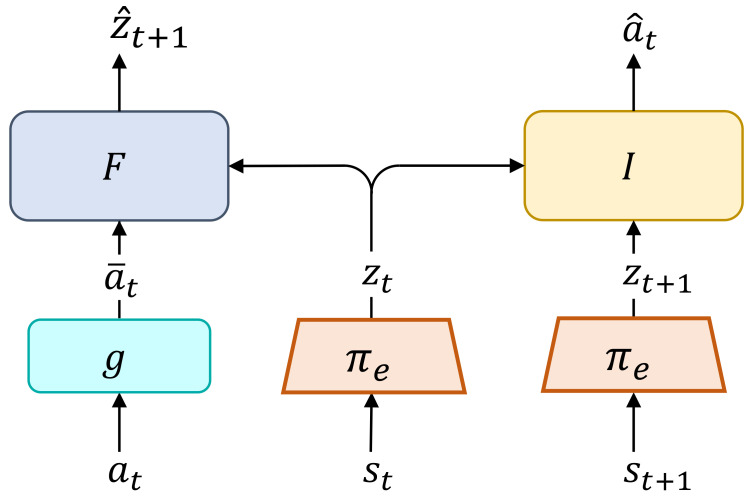
VPCPM for the encoder $\pi_{e}$: At the *forward* step, the forward model *F* takes the inputs as the latent state $z_{t}$ and the latent action ${\bar{a}}_{t}$ for predicting next latent state $z_{t+1}$. In this step, both *F* and $\pi_{e}$ are optimized together. At the *inverse* step, the inverse model *I* takes the inputs as two latent states $z_{t}$ and $z_{t+1}$ for predicting the action $a_{t}$. In this step, both *I* and $\pi_{e}$ are optimized together.

An overview of the proposed method is shown in Figure 2. The visual encoder $\pi_{e}:O\to Z$ learns the mapping from the observation into the latent space. VPCPM alternates between learning the forward dynamic model (*forward* step) and inverse dynamics model (*inverse* step) while optimizing the underlying encoder $\pi_{e}$ (Algorithm 1). At the *forward* step, the forward model *F* parameterized by ψ takes the inputs as the current latent state and the latent action in predicting the next latent state. To optimize *F* together with $\pi_{e}$, the InfoNCE loss [42] is employed, which contrasts between the predicted next latent state and the ground truth. Formally, let f:Z×Z→R be a similarity metric; the objective of forward model is described as:

(5) $$J_{F}\left(\psi ,\phi \right)=-\frac{1}{K}\sum_{i}\log\frac{\exp f({\hat{z}}_{t+1}^{\left(i\right)},{\bar{z}}_{t+1}^{\left(i\right)})}{\frac{1}{K}\sum_{j}\exp f({\hat{z}}_{t+1}^{\left(i\right)},{\bar{z}}_{t+1}^{\left(j\right)})},$$

where ${\hat{z}}_{t+1}^{\left(i\right)}=F\left(\pi_{e}\left(s_{t}^{\left(i\right)}\right),{\bar{a}}_{t}^{\left(i\right)};\psi \right)$ is the predicted latent state, and ${\bar{z}}_{t+1}^{\left(i\right)}=\pi_{e}\left(s_{t+1}^{\left(i\right)}\right)$ is the target latent state without encoder parameter update. The expectation is computed over *K* samples of $\left(s_{t},a_{t},s_{t+1}\right)$. Operating in the latent space bypasses the prediction of the forward model in pixel space, which would be extremely challenging given the large uncertainty in pixel prediction. The use of InfoNCE helps to learn the discriminative representation, where the dissimilar states are repelled and the similar states are pulled close. Additionally, this objective also prevents trivial collapsed solutions in which the constant features are obtained for every state.

**Algorithm 1** Visual pretraining via contrastive predictive model (VPCPM)
1:**Input**: Dataset $D={\left\{\left(s_{t}^{\left(i\right)},a_{t}^{\left(i\right)},s_{t+1}^{\left(i\right)}\right)\right\}}_{i=1}^{N}$, the encoder’s parameters *ϕ*, batch size *K*2:**Output**: The encoder’s parameters *ϕ*3:**Initialize:** The parameters *ϕ*, *ψ*, *ρ*4:**for***k* = 1 **to** ∞ **do**5:   Sample a batch $B={\left\{\left(s_{t}^{\left(i\right)},a_{t}^{\left(i\right)},s_{t+1}^{\left(i\right)}\right)\right\}}_{i=1}^{K}$6:   Compute the latent states:                  $z_{t}^{\left(i\right)}=\pi_{e}\left(AUG\left(s_{t}^{\left(i\right)}\right)\right)$                  
$z_{t+1}^{\left(i\right)}=\pi_{e}\left(AUG\left(s_{t+1}^{\left(i\right)}\right)\right)$7:   Train forward model: *J_F_*(*ψ*, *ϕ*)    ▹ Equation (5)8:   Train inverse model: *J_I_*(*ρ*, *ϕ*)             ▹ Equation (6)9:
**end for**



In the *inverse* step, the inverse model *I* parameterized by *ρ* takes the states before and after transition and predicts the action in between. In this work, the inverse model operates in the latent space extracted by the visual encoder $\pi_{e}$. The encoder is jointly optimized with the inverse model by minimizing the following objective:

(6) $$J_{I}\left(\rho ,\phi \right)=\frac{1}{K}\sum_{i}\ell \left(I\left(\pi_{e}\left(s_{t}^{\left(i\right)}\right),\pi_{e}\left(s_{t+1}^{\left(i\right)}\right);\rho \right),a_{t}^{\left(i\right)}\right).$$

For continuous action, *ℓ* can be defined as the mean squared error or mean absolute error between ground truth and the predicted action. When predicting the action, the inverse model pays attention to the controllable features and the temporal difference between consecutive states in latent space, which also encourages the encoder to capture the discriminative features. Pretraining the representations satisfying both forward and inverse dynamics models strengthens the relations of state and actions in latent space, establishing an initialization point of the encoder for further fine-tuning inside a region of parameter space in which the parameters are henceforth restricted.

## 5. Experimental Results

### 5.1. Experiment Setup

**Figure 3:**
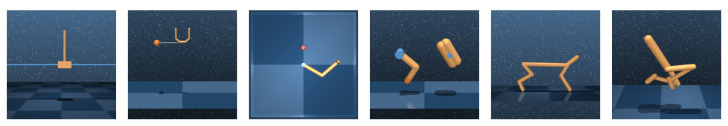
VPCPM is benchmarked on six image-based control environments from the DeepMind Control Suite [16]. The order of environment from lowest to the highest dimension of action: Cartpole, Ball in cup, Reacher, Finger, Cheetah, and Walker. Each task offers a unique set of challenges, including complex dynamics, sparse rewards, and hard exploration.

The proposed method is evaluated on a diverse set of the image-based control tasks from DeepMind Control suite [16], which was recently considered as a standard for benchmarking the sample efficiency of RL from images [14,17,77,78]. DMControl consists of different robot models (environments), where each model can be associated with a particular MDP representing a specific task. The selected benchmark includes six environments from the PlaNet benchmark [77], as shown in Figure 3, where the action repeat is treated as a hyperparameter (Table 1). The settings of visual observation follow [14,17,18,36], which consider a stack of three consecutive 84 × 84 RGB renderings as a state.

For the vision-based RL algorithm, we use two state-of-the-art methods including RAD [17] and DrQ [18], which are based on soft-actor critic (SAC) [75]. The network architecture used is identical to [17,18,36]. Unless stated otherwise, the configurations of the algorithm are as follows: the actor and critic neural network are trained using Adam optimizer [35] and a mini-batch size of 512. For SAC, the initial temperature is 0.1, the soft target update rate *τ* is 0.01, and the target network and the actor updates are performed every two critic updates similar [17,18,36]. The random cropping [14,17] is used as image augmentation during pretraining. The learning rate for actor, critic, and the parameter *α* of RAD and DrQ is followed by the setup from each method. In our CPM, the forward model is parameterized by four 50-d hidden layers followed by ReLU activation except the last, and the inverse model is parameterized by three 1024-d hidden layers followed by ReLUs, except the last layer, which uses tanh to normalize the actions. The action is encoded by an MLP consisting of two 50-d hidden layers followed by ReLU except for the last. Input to the forward model MLP is a concatenation of the current latent state and the current encoded action. Input to the inverse model MLP is a concatenation of the current and next latent states. Both the forward and inverse model have separated Adam optimizer [79]. During the pretraining stage, the encoder, inverse, and forward model are trained with a learning rate of 1 × 10^−4^ for Walker, 2 × 10^−4^ for Cheetah, and 1 × 10^−3^ otherwise, with the batch size of 512.

The performance of the agent is evaluated across five seeds; for every seed, the average returns of 10 episodes are computed every 10k environment steps. The figures plot the mean performance together with ±1 standard deviation shading. The performance is reported over the true environment steps as a common practice [14,17,18,36,78], thus are invariant to the action repeat hyperparameter. Throughout experiments, pretraining data are collected by a random policy. Specifically, for Cheetah and Walker domain, 50k transitions are collected, and 25k for the others. The encoder is pretrained within 50k iterations for Cheetah and Walker, and 25k otherwise, which corresponds to one update step per transition. The full set of parameters is shown in Table 1.

### 5.2. Effects of Pretrained Representation

In this section, the effectiveness of VPCPM in pretraining the visual encoder for different vision-based algorithms is investigated. Two state-of-the-art algorithms, including RAD [17] and DrQ [18], are evaluated, where the hyperparameters reported in each method are used. The random crop augmentation from each method is applied. Crop image augmentation in RAD is formed by cropping 84 × 84 frames from an input frame of 100 × 100, while in DrQ, the 84 × 84 frames are padded each side by ±4, then cropped back to the 84 × 84 size. Moreover, in the RAD paper, there are some tasks using translation augmentation; we instead use crop augmentation across tasks, thus the results may be varied. The parameters of the pretrained encoder are fine-tuned by the base RL algorithm in a specific task.

**Figure 4:**
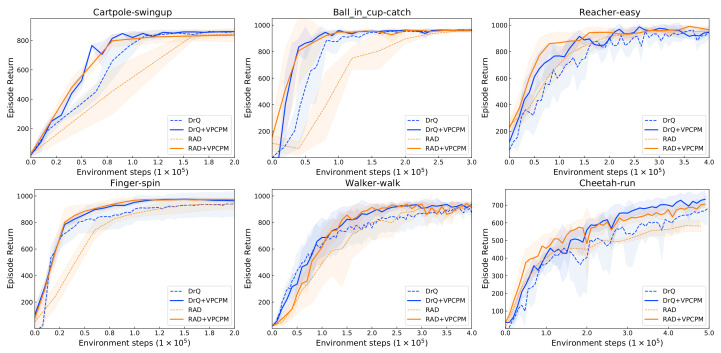
The performance on six tasks from PlaNet benchmark [77]. Pretraining the visual encoder by VPCPM consistently improves performance and sample efficiency across all environments.

Figure 4 and Table 2 compare these methods with and without pretraining. We provide the result at both 100k and 500k steps as common report for DMControl [14,17,18,46]. At 100k step, VPCPM enhances over RAD ranging from 13 to 118%, with the largest magnitude on Ball in cup-catch. For DrQ, the enhancements are in the range from 6 to 20%, with the largest magnitude on Reacher-easy. From Figure 4, the improvement is shown clearer at the early stage of training on the sparse reward tasks such as Ball in cup-catch and Finger-spin. The reason is that these tasks usually failed to complete the task at the beginning, thus observing less reward signal to learn the visual representation. With our VPCPM-initialized representations, the policy part can be quickly learned, resulting in significantly accelerating learning progress. For the Cartpole-swingup task, the action space is very small (with the dimension of one); thus, with the well-presented representation of states, the task can be quickly solved. The tasks including Reacher-easy, Walker-walk, and Cheetah-run are more challenging because of the exploration problem, thus requiring more samples to complete the tasks even with good representations, resulting in a lower magnitude of the improvement. Overall, our method improves 44% over RAD and 10% over DrQ at 100k steps. At 500k steps, the enhancement is smaller, with 3.4% over RAD and 2.9% over DrQ. This is because the base algorithms are almost converged around 500k; thus, the effect of pretraining is moderate.

### 5.3. Comparison with Prior Methods

In this section, the comparison of pretraining by using different unsupervised learning methods is conducted. VPCPM is compared against two representation learning approaches: non-model-based and model-based. The non-model-based representation learning approach includes (i) reconstruction loss as in VAE [35], and (ii) the contrastive loss from single observation as in CURL [14]. The model-based approach includes (iii) a simple predictive model (PM), where the forward and inverse model are learned by mean square error loss, (iv) augmented temporal contrast (ATC) [15], where the marginal forward model, i.e., without conditioning on actions, is parameterized by a residual network and learned using contrastive loss, and (v) predictive coding-consistency-curvature (PC3) [80], where the forward model is learned by the weighted sum of three losses: contrastive loss, mean square error loss, and low curvature loss. In PC3, the current latent state-action pair $\left(z_{t},a_{t}\right)$ is used as the source of negative samples and used for the contrastive prediction of the next latent state $z_{t+1}$. In contrast, we use the predicted next latent state ${\hat{z}}_{t+1}$ as the source of negative samples. For the implementation of ATC, we use our implementation with the modification as follows, disable the inverse model, and remove the action input of the forward model. For PC3, we use the author’s provided code (https://github.com/VinAIResearch/PC3-pytorch.git, accessed on 17 March 2022). For a fair comparison, the same amount of samples is used during pretraining. The procedure for evaluation is similar to the previous section, but only the RAD algorithm is considered.

**Figure 5:**
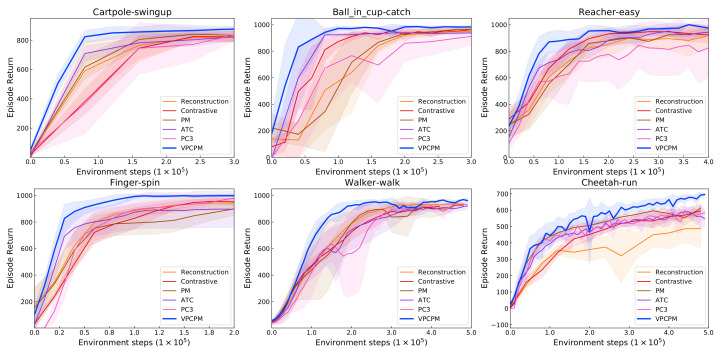
VPCPM compared to prior unsupervised learning methods. The improvement shows the importance of dynamics constraints during pretraining, in addition to the semantic information.

The performance in Figure 5 shows that RAD initialized by VPCPM outperforms across all environments. These improvements suggest the importance of imposing the dynamics to the visual encoder during pretraining, which is lacking in the methods that only focus on semantic information such as reconstruction and contrastive. In comparison to the simple PM, the proposed method benefits from the contrastive objective. Indeed, the inverse model is limited since it cannot capture the changes in the sensory stream beyond the agent’s control, and the use of contrastive is helpful to prevent this degeneracy. Moreover, learning in a contrastive manner represents states more discriminative in the latent space. Compared with ATC, VPCPM shows the importance of the action-condition forward model together with the inverse model in learning the controllable features. PC3 is originally designed to use for model-based planning algorithms such as iLQR, which requires the system to be locally linear. Thus, the features learned by PC3 might not suitable for vision-based RL algorithms in the highly nonlinear system, as in our considered environments. Indeed, the results show that the representations from VPCPM are more useful for vision-based RL algorithms. Overall, the sample efficiency in deep RL should be attained from representations that are discriminative and follow the dynamics.

### 5.4. Effects of Components during Pretraining

**Figure 6:**
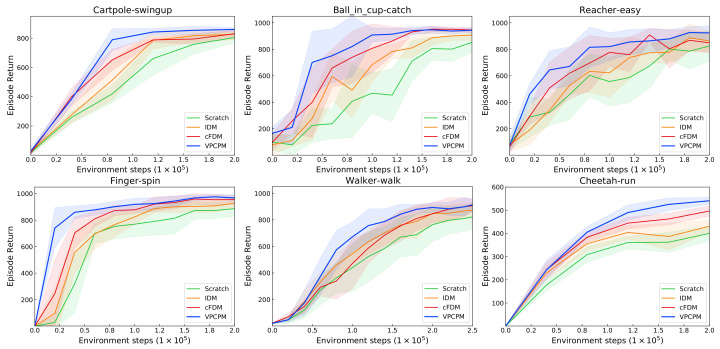
The effectiveness of each component in VPCPM. Learning the encoder together with contrastive FDM (cFDM) shows more effectiveness than with IDM. Overall, the cFDM in conjunction with IDM forms better representation, thus better performance.

Ablation tests were performed to determine the effects of individual components in VPCPM. The performances of RAD with the encoder pretrained by using the contrastive forward dynamic model (cFDM), inverse dynamic model (IDM), and both of them (VPCPM) are shown in Figure 6. Overall, the base RL agent benefits from pretraining by any type of dynamic model, but the cFDM shows more impacts. Together with the constraint of IDM, the proposed method significantly accelerates the sample efficiency of the base algorithm. Moreover, training the visual encoder together with FDM purely in latent space does not suffer from the collapsed problem, where the encoder outputs a constant across states.

### 5.5. Generalization over Unseen Tasks

**Figure 7:**
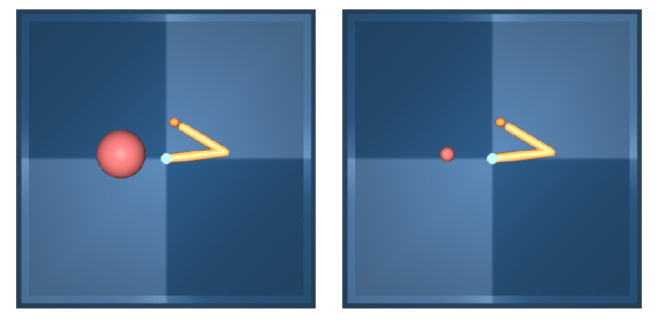
An example of observation from Reacher-easy (**left**) and Reacher-hard (**right**). The different size of the goal indicator causes the degraded performance when freezing the encoder. However, fine-tuning from this encoder still shows higher performance compared to training from scratch.

In this section, the generalization of the pretrained encoder for unseen tasks is examined. Specifically, the encoder pretrained from a source task is used for the unseen target tasks. Subsequently, the RAD agent is trained on top of the pretrained encoder until converged. The considered tasks are shown in Table 3. The target tasks are different on the reward function but share the same observation space, except for Reacher-hard, where the size of the visual indicator is different (see Figure 7). The performance of the base agent is evaluated in both “fine-tuning” and “frozen” settings, where the pretrained representation is frozen or fine-tuned. The results are averaged across five seeds and compared against the agent learning from scratch.

**Figure 8:**
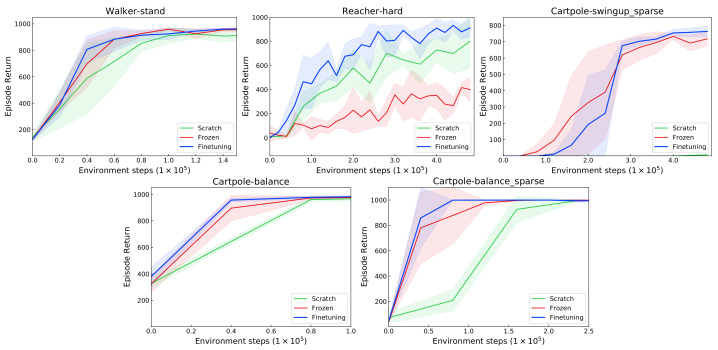
Performance on unseen tasks comes from the same domain as the source task. The pretrained representation enables the RL agent to significantly improve sample efficiency, especially in sparse reward tasks.

The results are shown in Figure 8. In almost all tasks, the frozen representation is sufficient in learning optimal policy. When fine-tuning from the pretrained initialization, the performance is slightly improved. The major exception is the Reacher-hard task, where the frozen encoder significantly underperforms. However, the fine-tuning encoder shows more sample efficiency than that learning from scratch. The difference in the observation space causes this downgraded performance, i.e., the different size of the target indicator. The enhancement in the performance of the base RL agent shows that VPCPM is successfully learning the abstract features without reward supervision.

### 5.6. Pretraining with Classification

**Figure 9:**
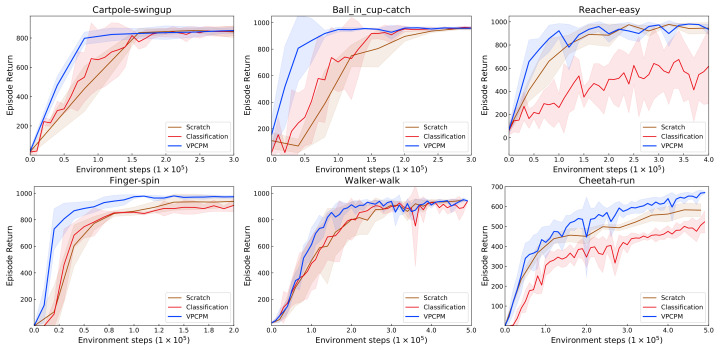
The comparison of training from scratch, pretraining by classification, and VPCPM. The results show that the classification task does not lead to improved sample efficiency.

To show the importance of imposing the dynamics in representation learning, we investigate the case where the visual encoder is trained to capture the semantic only, without knowing about the dynamics. To investigate that, we consider six-way classification which corresponds to six robotic models from DeepMind Control Suite [16], as indicated in Figure 3. The dataset is generated by an expert policy. In each class, the training and test sets contain 50k and 10k samples, respectively. In total, there are 300k samples for training and 60k samples for testing. The visual encoder and the classifier are trained using Adam optimizer [79] with the learning rate 3 × 10^−4^, and *β* = (0.9, 0.999). We use the data augmentation methods used in [43] and the random crop [17]. The pretrained encoder is then frozen and used for policy learning. The results are shown in Figure 9. In Ball in cup-catch and Cartpole-swingup, pretraining by classification slightly improves performance, while other tasks show no gain, or even degraded performance. The results indicate the importance of making the representation encode dynamic information for learning representation offline for RL tasks.

## 6. Conclusions

In this paper, a new self-supervised representation learning method is proposed to pretrain the visual encoder for the vision-based RL. By leveraging plenty of reward-free data, the proposed method successfully learns the meaningful initial representations that provide sufficient information and consistently follow underlying dynamics from an environment. Experimental results show that the state-of-the-art vision-based RL algorithms benefit from our method, with the gain of 44% over RAD and 10% over DrQ at 100k steps. Additionally, we benchmark several leading self-supervised methods for pretraining visual encoders. The results show that the performance of the policy learned on top of the VPCPM-trained encoder matches or outperforms all others. Furthermore, the independence of task-specific rewards during pretraining allows our learned representations to be reused for different tasks sharing similar observation and action space.

In this paper, we investigate the effectiveness of pretraining the visual encoder, in which the testing and training environments are similar. However, this condition is brittle in practice. Future works should improve the robustness of the pretrained representation such that it is invariant to the visual distractions from the environment such as variations in background, color, and camera pose.

## Figures and Tables

**Table 1:** Hyperparameters for training the vision-based RL algorithms: RAD [17] and DrQ [18].

Parameter	RAD [17]	DrQ [18]
Action repeat	2 Finger, spin; Walker, walk	
	8 Cartpole, swingup	
	4 otherwise	
Batch size	512	512
Augmentation	Crop [17]	Crop [18]
Replay buffer size	10^5^	10^5^
Initial steps	1000	1000
Stacked frames	3	3
Discount *γ*	0.99	0.99
Optimizer	Adam	Adam
Learning rate (*π_e_*, *π_a_*, *Q*)	2 × 10^−4^ cheetah	1× 10^−3^ (all)
	1 × 10^−3^ otherwise	
Learning rate (*α*)	1 × 10^−4^	1 × 10^−4^
Critic target update frequency	2	2
Critic soft-update rate *τ*	0.01	0.01
Actor update frequency	2	2
Actor log stddev bounds	[−10, 2]	[−10, 2]
Initial temperature	0.1	0.1

**Table 2:** Episodic return at 100k and 500k environment steps with mean and standard deviation for 5 seeds. The scores are bold, indicating the improvement compared to the corresponding base RL algorithm.

	RAD [17]		DrQ [18]	
100k Steps	Scratch	+VPCPM	Scratch	+VPCPM
Finger, spin	860 ± 29	**974 ± 15**	901 ± 104	**958 ± 20**
Cartpole, swingup	454 ± 155	**798 ± 42**	759 ± 92	**825 ± 40**
Reacher, easy	704 ± 212	**855 ± 117**	601 ± 213	**720 ± 65**
Cheetah, run	365 ± 31	**420 ± 81**	344 ± 67	**384 ± 45**
Walker, walk	493 ± 175	**614 ± 128**	612 ± 164	**651 ± 115**
Ball in cup, catch	421 ± 247	**918 ± 25**	913 ± 53	**959 ± 7**
500k steps				
Finger, spin	982 ± 2	**985 ± 15**	938 ± 103	**988 ± 5**
Cartpole, swingup	867 ± 10	**870 ± 13**	868 ± 10	**877 ± 11**
Reacher, easy	945 ± 34	**965 ± 41**	942 ± 71	**945 ± 43**
Cheetah, run	573 ± 35	**670 ± 7**	660 ± 96	**698 ± 47**
Walker, walk	948 ± 11	**952 ± 15**	921 ± 45	**960 ± 10**
Ball in cup, catch	962 ± 5	**964 ± 13**	963 ± 9	**968 ± 4**

**Table 3:** The encoder pretrained with data from a source task is reused for new target tasks. Almost the encoders are successfully generalized to target tasks, except for Reacher-hard, needing to fine-tune.

Source Task	Target Task
Cartpole-swingup	Cartpole-swingup_sparse
	Cartpole-balance
	Cartpole-balance_sparse
Walker-walk	Walker-stand
Reacher-easy	Reacher-hard

## Data Availability

Not applicable.

## References

[1] Mnih V., Kavukcuoglu K., Silver D., Rusu A.A., Veness J., Bellemare M.G., Graves A., Riedmiller M., Fidjeland A.K., Ostrovski G. (2015). Human-level control through deep reinforcement learning. Nature.

[2] Jaderberg M., Mnih V., Czarnecki W.M., Schaul T., Leibo J.Z., Silver D., Kavukcuoglu K. Reinforcement learning with unsupervised auxiliary tasks. Proceedings of the 5th International Conference on Learning Representations, ICLR 2017.

[3] Espeholt L., Soyer H., Munos R., Simonyan K., Mnih V., Ward T., Doron Y., Firoiu V., Harley T., Dunning I. Impala: Scalable distributed deep-rl with importance weighted actor-learner architectures. Proceedings of the 35th International Conference on Machine Learning, ICML 2018.

[4] Levine S., Finn C., Darrell T., Abbeel P. (2016). End-to-end training of deep visuomotor policies. J. Mach. Learn. Res..

[5] Lee A.X., Nagabandi A., Abbeel P., Levine S. Stochastic latent actor-critic: Deep reinforcement learning with a latent variable model. Proceedings of the 34th Advances in Neural Information Processing Systems, NeurIPS 2020.

[6] Kalashnikov D., Irpan A., Pastor P., Ibarz J., Herzog A., Jang E., Quillen D., Holly E., Kalakrishnan M., Vanhoucke V. Scalable deep reinforcement learning for vision-based robotic manipulation. Proceedings of the 2nd Conference on Robot Learning, CoRL 2018.

[7] Akkaya I., Andrychowicz M., Chociej M., Litwin M., McGrew B., Petron A., Paino A., Plappert M., Powell G., Ribas R. (2019). Solving rubik’s cube with a robot hand. arXiv.

[8] Julian R., Swanson B., Sukhatme G.S., Levine S., Finn C., Hausman K. Never Stop Learning: The Effectiveness of Fine-Tuning in Robotic Reinforcement Learning. Proceedings of the 4th Conference on Robot Learning, CoRL 2020.

[9] Liu H., Abbeel P. Behavior from the void: Unsupervised active pre-training. Proceedings of the 35th Advances in Neural Information Processing Systems, NeurIPS 2021.

[10] Shah R., Kumar V. Rrl: Resnet as representation for reinforcement learning. Proceedings of the 38th International Conference on Machine Learning, ICML 2021.

[11] Lange S., Riedmiller M. Deep auto-encoder neural networks in reinforcement learning. Proceedings of the International Joint Conference on Neural Networks, IJCNN 2010.

[12] Finn C., Tan X.Y., Duan Y., Darrell T., Levine S., Abbeel P. Deep spatial autoencoders for visuomotor learning. Proceedings of the International Conference on Robotics and Automation, ICRA 2016.

[13] Nair A.V., Pong V., Dalal M., Bahl S., Lin S., Levine S. Visual reinforcement learning with imagined goals. Proceedings of the 32th Advances in Neural Information Processing Systems, NeurIPS 2018.

[14] Srinivas A., Laskin M., Abbeel P. Curl: Contrastive unsupervised representations for reinforcement learning. Proceedings of the 37th International Conference on Machine Learning, ICML 2020.

[15] Stooke A., Lee K., Abbeel P., Laskin M. Decoupling representation learning from reinforcement learning. Proceedings of the 38th International Conference on Machine Learning, ICML 2021.

[16] Tunyasuvunakool S., Muldal A., Doron Y., Liu S., Bohez S., Merel J., Erez T., Lillicrap T., Heess N., Tassa Y. (2020). dm_control: Software and tasks for continuous control. Softw. Impacts.

[17] Laskin M., Lee K., Stooke A., Pinto L., Abbeel P., Srinivas A. Reinforcement Learning with Augmented Data. Proceedings of the 34th Advances in Neural Information Processing Systems, NeurIPS 2020.

[18] Yarats D., Kostrikov I., Fergus R. Image Augmentation Is All You Need: Regularizing Deep Reinforcement Learning from Pixels. Proceedings of the 9th International Conference on Learning Representations, ICLR 2021.

[19] Krizhevsky A., Sutskever I., Hinton G.E. Imagenet classification with deep convolutional neural networks. Proceedings of the 26th Advances in Neural Information Processing Systems, NeurIPS 2012.

[20] Wojke N., Bewley A., Paulus D. Simple online and realtime tracking with a deep association metric. Proceedings of the 24th IEEE International Conference on Image Processing, ICIP 2017.

[21] Wojke N., Bewley A. Deep cosine metric learning for person re-identification. Proceedings of the IEEE Winter Conference on Applications of Computer Vision, WACV 2018.

[22] Peng Y., Rysanek A., Nagy Z., Schlüter A. (2018). Using machine learning techniques for occupancy-prediction-based cooling control in office buildings. Appl. Energy.

[23] He K., Gkioxari G., Dollár P., Girshick R. Mask r-cnn. Proceedings of the IEEE International Conference on Computer Vision, ICCV 2017.

[24] Vu T., Jang H., Pham T.X., Yoo C. Cascade rpn: Delving into high-quality region proposal network with adaptive convolution. Proceedings of the 33rd Advances in Neural Information Processing Systems, NeurIPS 2019.

[25] Vu T., Kang H., Yoo C.D. Scnet: Training inference sample consistency for instance segmentation. Proceedings of the 35th Association for the Advancement of Artificial Intelligence, AAAI 2021.

[26] Jiang L., Zhao H., Shi S., Liu S., Fu C.W., Jia J. Pointgroup: Dual-set point grouping for 3d instance segmentation. Proceedings of the IEEE/CVF Computer Vision and Pattern Recognition, CVPR 2020.

[27] Chen S., Fang J., Zhang Q., Liu W., Wang X. Hierarchical aggregation for 3d instance segmentation. Proceedings of the IEEE/CVF International Conference on Computer Vision, ICCV 2021.

[28] Vu T., Kim K., Luu T.M., Nguyen X.T., Yoo C.D. SoftGroup for 3D Instance Segmentation on 3D Point Clouds. Proceedings of the IEEE/CVF Computer Vision and Pattern Recognition, CVPR 2022.

[29] Choy C.B., Xu D., Gwak J., Chen K., Savarese S. 3d-r2n2: A unified approach for single and multi-view 3d object reconstruction. Proceedings of the 14th European Conference on Computer Vision, ECCV 2016.

[30] Rosinol A., Sattler T., Pollefeys M., Carlone L. Incremental visual-inertial 3d mesh generation with structural regularities. Proceedings of the IEEE International Conference on Robotics and Automation, ICRA 2019.

[31] Chen M., Tang Y., Zou X., Huang K., Li L., He Y. (2019). High-accuracy multi-camera reconstruction enhanced by adaptive point cloud correction algorithm. Opt. Lasers Eng..

[32] Zhang F., Leitner J., Milford M., Upcroft B., Corke P. (2015). Towards vision-based deep reinforcement learning for robotic motion control. arXiv.

[33] Ebert F., Finn C., Dasari S., Xie A., Lee A., Levine S. (2018). Visual foresight: Model-based deep reinforcement learning for vision-based robotic control. arXiv.

[34] Beattie C., Leibo J.Z., Teplyashin D., Ward T., Wainwright M., Küttler H., Lefrancq A., Green S., Valdés V., Sadik A. (2016). Deepmind lab. arXiv.

[35] Kingma D.P., Welling M. (2013). Auto-encoding variational bayes. arXiv.

[36] Yarats D., Zhang A., Kostrikov I., Amos B., Pineau J., Fergus R. Improving sample efficiency in model-free reinforcement learning from images. Proceedings of the 35th Association for the Advancement of Artificial Intelligence, AAAI 2021.

[37] Agrawal P., Nair A.V., Abbeel P., Malik J., Levine S. Learning to poke by poking: Experiential learning of intuitive physics. Proceedings of the 30th Advances in Neural Information Processing Systems, NeurIPS 2016.

[38] Pathak D., Agrawal P., Efros A.A., Darrell T. Curiosity-driven exploration by self-supervised prediction. Proceedings of the 34th International Conference on Machine Learning, ICML 2017.

[39] Pathak D., Mahmoudieh P., Luo G., Agrawal P., Chen D., Shentu Y., Shelhamer E., Malik J., Efros A.A., Darrell T. Zero-shot visual imitation. Proceedings of the IEEE/CVF Computer Vision and Pattern Recognition Workshop, CVPR 2018.

[40] Böhmer W., Springenberg J.T., Boedecker J., Riedmiller M., Obermayer K. (2015). Autonomous learning of state representations for control: An emerging field aims to autonomously learn state representations for reinforcement learning agents from their real-world sensor observations. Künstl. Intell..

[41] Lesort T., Díaz-Rodríguez N., Goudou J.F., Filliat D. (2018). State representation learning for control: An overview. Neural Netw..

[42] Oord A.v.d., Li Y., Vinyals O. (2018). Representation learning with contrastive predictive coding. arXiv.

[43] He K., Fan H., Wu Y., Xie S., Girshick R. Momentum contrast for unsupervised visual representation learning. Proceedings of the TEEE/CVF Computer Vision and Pattern Recognition, CVPR 2020.

[44] Chen T., Kornblith S., Norouzi M., Hinton G. A simple framework for contrastive learning of visual representations. Proceedings of the 37th International Conference on Machine Learning, ICML 2020.

[45] Schwarzer M., Anand A., Goel R., Hjelm R.D., Courville A., Bachman P. Data-efficient reinforcement learning with self-predictive representations. Proceedings of the 9th International Conference on Learning Representations, ICLR 2021.

[46] Lee K.H., Fischer I., Liu A., Guo Y., Lee H., Canny J., Guadarrama S. Predictive information accelerates learning in rl. Proceedings of the 34th Advances in Neural Information Processing Systems, NeurIPS 2020.

[47] Mnih V., Badia A.P., Mirza M., Graves A., Lillicrap T., Harley T., Silver D., Kavukcuoglu K. Asynchronous methods for deep reinforcement learning. Proceedings of the 33rd International Conference on Machine Learning, ICML 2016.

[48] Bellemare M.G., Naddaf Y., Veness J., Bowling M. (2013). The arcade learning environment: An evaluation platform for general agents. J. Artif. Intell. Res..

[49] Anand A., Racah E., Ozair S., Bengio Y., Côté M.A., Hjelm R.D. Unsupervised state representation learning in atari. Proceedings of the 33rd Advances in Neural Information Processing Systems, NeurIPS 2019.

[50] Grill J.B., Strub F., Altché F., Tallec C., Richemond P.H., Buchatskaya E., Doersch C., Pires B.A., Guo Z.D., Azar M.G. Bootstrap your own latent: A new approach to self-supervised learning. Proceedings of the 34th Advances in Neural Information Processing Systems, NeurIPS 2020.

[51] Fischer I. (2020). The conditional entropy bottleneck. Entropy.

[52] Zhang A., McAllister R., Calandra R., Gal Y., Levine S. Learning invariant representations for reinforcement learning without reconstruction. Proceedings of the 9th International Conference on Learning Representations, ICLR 2021.

[53] Agarwal R., Machado M.C., Castro P.S., Bellemare M.G. Contrastive behavioral similarity embeddings for generalization in reinforcement learning. Proceedings of the 9th International Conference on Learning Representations, ICLR 2021.

[54] Ferns N., Precup D. Bisimulation Metrics are Optimal Value Functions. Proceedings of the 30th Association for Uncertainty in Artificial Intelligence, UAI 2014.

[55] Radford A., Narasimhan K., Salimans T., Sutskever I. (2018). Improving Language Understanding by Generative Pre-Training. https://openai.com/blog/language-unsupervised.

[56] Radford A., Wu J., Child R., Luan D., Amodei D., Sutskever I. Language models are unsupervised multitask learners. OpenAI Blog 2019. https://d4mucfpksywv.cloudfront.net/better-language-models/language-models.pdf.

[57] Devlin J., Chang M.W., Lee K., Toutanova K. Bert: Pre-training of deep bidirectional transformers for language understanding. Proceedings of the 17th North American Chapter of the Association for Computational Linguistics, NAACL 2019.

[58] Zbontar J., Jing L., Misra I., LeCun Y., Deny S. Barlow twins: Self-supervised learning via redundancy reduction. Proceedings of the 38th International Conference on Machine Learning, ICML 2021.

[59] Bardes A., Ponce J., LeCun Y. Vicreg: Variance-invariance-covariance regularization for self-supervised learning. Proceedings of the 10th International Conference on Learning Representations, ICLR 2022.

[60] Devin C., Abbeel P., Darrell T., Levine S. Deep object-centric representations for generalizable robot learning. Proceedings of the IEEE International Conference on Robotics and Automation, ICRA 2018.

[61] Pathak D., Gandhi D., Gupta A. Self-supervised exploration via disagreement. Proceedings of the 36th International Conference on Machine Learning, ICML 2019.

[62] Burda Y., Edwards H., Storkey A., Klimov O. Exploration by random network distillation. Proceedings of the 7th International Conference on Learning Representations, ICLR 2019.

[63] Aubret A., Matignon L., Hassas S. (2019). A survey on intrinsic motivation in reinforcement learning. arXiv.

[64] Nguyen T., Luu T.M., Vu T., Yoo C.D. Sample-efficient reinforcement learning representation learning with curiosity contrastive forward dynamics model. Proceedings of the IEEE/RSJ International Conference on Intelligent Robots and Systems, IROS 2021.

[65] Laskin M., Yarats D., Liu H., Lee K., Zhan A., Lu K., Cang C., Pinto L., Abbeel P. URLB: Unsupervised reinforcement learning benchmark. Proceedings of the 35th Advances in Neural Information Processing Systems, NeurIPS 2021.

[66] Yarats D., Fergus R., Lazaric A., Pinto L. Reinforcement learning with prototypical representations. Proceedings of the 38th International Conference on Machine Learning, ICML 2021.

[67] Lee L., Eysenbach B., Parisotto E., Xing E., Levine S., Salakhutdinov R. (2019). Efficient exploration via state marginal matching. arXiv.

[68] Eysenbach B., Gupta A., Ibarz J., Levine S. Diversity is all you need: Learning skills without a reward function. Proceedings of the 6th International Conference on Learning Representations, ICLR 2018.

[69] Hansen S., Dabney W., Barreto A., Van de Wiele T., Warde-Farley D., Mnih V. Fast task inference with variational intrinsic successor features. Proceedings of the 8th International Conference on Learning Representations, ICLR 2020.

[70] Liu H., Abbeel P. Aps: Active pretraining with successor features. Proceedings of the 38th International Conference on Machine Learning, ICML 2021.

[71] Singh H., Misra N., Hnizdo V., Fedorowicz A., Demchuk E. (2003). Nearest neighbor estimates of entropy. Am. J. Math. Manag. Sci..

[72] Caron M., Misra I., Mairal J., Goyal P., Bojanowski P., Joulin A. Unsupervised learning of visual features by contrasting cluster assignments. Proceedings of the 34th Advances in Neural Information Processing Systems, NeurIPS 2020.

[73] Sutton R.S., Barto A.G. (1998). Reinforcement Learning: An Introduction.

[74] Kaelbling L.P., Littman M.L., Cassandra A.R. (1998). Planning and acting in partially observable stochastic domains. Artif. Intell..

[75] Haarnoja T., Zhou A., Abbeel P., Levine S. Soft actor-critic: Off-policy maximum entropy deep reinforcement learning with a stochastic actor. Proceedings of the 35th International Conference on Machine Learning, ICML 2018.

[76] Ziebart B.D. (2010). Modeling Purposeful Adaptive Behavior with the Principle of Maximum Causal Entropy.

[77] Hafner D., Lillicrap T., Fischer I., Villegas R., Ha D., Lee H., Davidson J. Learning latent dynamics for planning from pixels. Proceedings of the International Conference on Machine Learning, ICML 2019.

[78] Hafner D., Lillicrap T., Ba J., Norouzi M. Dream to Control: Learning Behaviors by Latent Imagination. Proceedings of the 8th International Conference on Learning Representations, ICLR 2020.

[79] Kingma D.P., Ba J. Adam: A Method for Stochastic Optimization. Proceedings of the 5th International Conference on Learning Representations, ICLR 2014.

[80] Shu R., Nguyen T., Chow Y., Pham T., Than K., Ghavamzadeh M., Ermon S., Bui H. Predictive coding for locally-linear control. Proceedings of the 37th International Conference on Machine Learning, ICML 2020.

